# Improving the clinical management of traumatic brain injury through the pharmacokinetic modeling of peripheral blood biomarkers

**DOI:** 10.1186/s12987-016-0045-y

**Published:** 2016-11-30

**Authors:** Aaron Dadas, Jolewis Washington, Nicola Marchi, Damir Janigro

**Affiliations:** 1 Flocel Inc., Cleveland, OH 44103 USA; 2 The Ohio State University, Columbus, OH USA; 3 John Carroll University, University Heights, OH USA; 4 Laboratory of Cerebrovascular Mechanisms of Brain Disorders, Institut de Génomique Fonctionnelle, Université Montpellier, Montpellier, France; 5 Case Western Reserve University, Cleveland, OH USA

**Keywords:** Physiologically-based pharmacokinetic model, Precision medicine, Traumatic brain injury, Glomerular filtration, Serum markers

## Abstract

**Background:**

Blood biomarkers of neurovascular damage are used clinically to diagnose the presence severity or absence of neurological diseases, but data interpretation is confounded by a limited understanding of their dependence on variables other than the disease condition itself. These include half-life in blood, molecular weight, and marker-specific biophysical properties, as well as the effects of glomerular filtration, age, gender, and ethnicity. To study these factors, and to provide a method for markers’ analyses, we developed a kinetic model that allows the integrated interpretation of these properties.

**Methods:**

The pharmacokinetic behaviors of S100B (monomer and homodimer), Glial Fibrillary Acidic Protein and Ubiquitin C-Terminal Hydrolase L1 were modeled using relevant chemical and physical properties; modeling results were validated by comparison with data obtained from healthy subjects or individuals affected by neurological diseases. Brain imaging data were used to model passage of biomarkers across the blood–brain barrier.

**Results:**

Our results show the following: (1) changes in biomarker serum levels due to age or disease progression are accounted for by differences in kidney filtration; (2) a significant change in the brain-to-blood volumetric ratio, which is characteristic of infant and adult development, contributes to variation in blood concentration of biomarkers; (3) the effects of extracranial contribution at steady-state are predicted in our model to be less important than suspected, while the contribution of blood–brain barrier disruption is confirmed as a significant factor in controlling markers’ appearance in blood, where the biomarkers are typically detected; (4) the contribution of skin to the marker S100B blood levels depends on a direct correlation with pigmentation and not ethnicity; the contribution of extracranial sources for other markers requires further investigation.

**Conclusions:**

We developed a multi-compartment, pharmacokinetic model that integrates the biophysical properties of a given brain molecule and predicts its time-dependent concentration in blood, for populations of varying physical and anatomical characteristics. This model emphasizes the importance of the blood–brain barrier as a gatekeeper for markers’ blood appearance and, ultimately, for rational clinical use of peripherally-detected brain protein.

**Electronic supplementary material:**

The online version of this article (doi:10.1186/s12987-016-0045-y) contains supplementary material, which is available to authorized users.

## Background

Peripheral biomarkers have myriad potential uses for prognostication, treatment and pharmacovigilance in many diseases, including those of neurological nature. For example, levels of the brain-derived glial fibrillary acidic protein (GFAP), S100B, tau and Ubiquitin C-Terminal Hydrolase L1 (UCHL-1) in biological fluids have been shown to correlate with presence and severity of many neurological disorders. Steady-state blood levels of these biomarkers are measurable, albeit at low concentrations, and increase rapidly after head injury. The most common use for peripheral biomarkers has been in the field of traumatic brain injury (TBI). The possibility of using serum S100B as a diagnostic tool for patients with mild head injury (MHI) was first reported in 1995 [1]. It was first thought that S100B release was a biomarker of subtle brain damage after MHI, although data suggests that an equally relevant mechanism may involve the release of S100B through a disrupted blood–brain barrier (BBB), without necessarily involving actual cellular damage [2–5]. Comparable results were obtained with GFAP and UCHL-1 [6] which suggests that these markers also appear in blood when the BBB is compromised.

The brain parenchyma is protected by a vascular barrier, referred to as BBB. The system of capillaries forming the human BBB has approximately 20 m^2^ of exchange surface with brain tissue, and is separated from neurons by only a few microns. The BBB maintains a strict compartmentalization of brain-and-blood-specific substances through the presence of a tight-junctioned endothelial cell layer. During blood–brain barrier disruption (BBBD), proteins normally present in high concentrations in the CNS are free to diffuse into the blood following their concentration gradients [7]. An ideal and clinically significant biomarker should be: (1) present at low or undetectable levels in serum of normal subjects under steady-state conditions; (2) present in brain and cerebrospinal fluid (CSF) at higher concentrations than in blood; (3) susceptible to extravasation in the event of BBBD; (4) further released by brain cells in response to brain damage (e.g., during reactive gliosis).

Among the several reasons that made the use of brain biomarkers a holy grail for neurology is the minimally-invasive nature of the process required to obtain blood samples. While a venipuncture is typically required, such a procedure is clearly less morbid than CSF sampling or the use of intravascular contrast agents (e.g., gadolinium or iodinated contrast agents). In addition, imaging modalities such as computed tomography (CT) scans expose the patient to radiation. Last, but perhaps not least, is the cost differential between state-of-the-art medical imaging and a simple blood test.

While the advantages of peripheral biomarkers are well understood, their widespread use has been confounded by several factors including inter-individual variability in “reference values”, the effect of age on markers’ presence or levels, ethnic differences, etc. Many groups have described this variability, and most of the data presented so far has focused on the astrocytic protein S100B [8]. This biomarker has been studied for several years, and investigated as a tool to diagnose non-CNS conditions, mainly malignant melanoma [9]. Other markers are being investigated, and it’s very likely that a number of new markers will become available in the next decade. We hypothesize that one of the obstacles in the acceptance of peripheral biomarker detection as a diagnostic approach for neurological diseases is the lack of understanding on how serum biomarker levels are holistically controlled by other physiological functions and parameters. For example, it has been suggested that S100B levels directly depend on body mass index (BMI) [10], while others have suggested that the increased BBB permeability in diabetes or conditions associated with obesity are the underlying factors contributing to this variability [11]. We developed a computer model that mimics, for a range of biomarker proteins, the key physiological features (e.g., BBB permeability, extracranial contribution) and pharmacokinetic properties (e.g., biomarker size and distribution, renal elimination) that contribute to changes in serum biomarker levels irrespective of neurological triggers.

## Methods

### Literature review for initial assignments of the model

The following sources were used to obtain the quantitative values used as initial conditions for our model. Values for total blood volume (TBV) were calculated using Nadler’s formula shown in Eq. 1:

1 $$ {\text{TBV}},{\text{ male }} = \, \left( {0. 3 6 6 9 { }*{\text{ height}}} \right) \, + \, \left( {0.0 3 2 1 9 { }*{\text{ weight}}} \right) \, + \, 0. 60 4 1 $$

$$ {\text{TBV}},{\text{ female }} = \, \left( {0. 3 5 6 1 { }*{\text{ height}}} \right) \, + \, \left( {0.0 3 30 8 { }*{\text{ weight}}} \right) \, + \, 0. 1 8 3 3 $$

where height and weight must be in units of meter and kilogram but are considered in the formula as unit-less quantities. Values for kidney function were acquired from [12]. Initial biomarker levels in brain were obtained from sources identified in Table 1. The values for maximal leakage of S100B and its homodimer were derived from our previous work [4, 11, 13]. The quantitative assignment of S100B levels in the human tissue of peripheral organs was similarly based on previous data [11]. Glomerular filtration rate (GFR) was calculated using the Cockcroft–Gault formula shown in Eq. 2:

**Table 1:** Initial parameter values used within model

Model feature	Parameter	Value	References
Brain biomarker concentration	S100b monomer (10.7 kD)	10.0 ng/ml = 1.0 nM	[23, 25, 36, 37]
	S100b dimer (21.0 kD)	10.0 ng/ml = 0.5 nM	
	GFAP (26.0 kD)	1.0 ng/ml = 0.038 nM	
	UCHL-1 (26.0 kD)	7.6 ng/ml = 0.292 nM	
Blood–brain barrier	Steady-state, newborn	10% of maximal BBBD	[4, 11, 13]
	Steady-state, adult	1–5% of maximal BBBD	
Central nervous system	Brain volume, newborn	0.42 l	[38]
	Brain volume, adult	1.42 l (male) 1.05 l (female)	[15, 16]
	Blood volume, newborn	0.28 l	[39]
	Blood volume, adult	6.0 l	[15, 16]
Skin	Skin volume, adult	7.8 l	[15]
	S100b in light skin	0.288 ng/ml	[11]
	S100b in dark skin	2.0 ng/ml	
Kidneys	Glomerular filtration rate	GFR (ml/min) = ((A*((SrCr/B)) ^ 1.209) * (0.993 ^ Age)	[12]
	Coefficient A (Caucasian)	141 (male) 144 (female)	
	Coefficient A (African American)	163 (male) 166 (female)	
	Coefficient B	0.9 (male) 0.7 (female)	

2 $$ {\text{GFR }} = \, \left( {{\text{GFR}}_{\text{Function}} * \, \left( {{\text{A }}* \, \left( {\left( {{\text{SrCr}}/{\text{B}}} \right)^{ \exp } } \right) \, * \, \left( {0. 9 9 3^{\text{age}} } \right)} \right) \, *{ 6}0} \right) $$

where the variables A, B, exp and serum creatinine (SrCr) are race-and-gender-dependent, and GFR_Function_ ranges from 0 to 1 and is indicative of kidney health. Due to the nature of the Cockcroft–Gault formula, changing age does little to influence the outcome of the model; as such, the value of Age was standardized to 45 years.

### Physiologically-based biomarker kinetic model development

Our model was developed using the SimBiology extension of MatLab (MathWorks, Natick MA), and results were analyzed in the Origin Pro 9.0 (Northampton, MA) and JMP 11 (SAS) programs. Our methods were derived from a generic Physiologically-Based Pharmacokinetic (PBPK) model developed by others [14], which was further based on a previous multi-compartment system in which several organs were represented with realistic dimensions [15, 16]. In the aforementioned model, the organs were connected by arterial and venous circulation with appropriate hemodynamic values, also obtained from the literature. For the model described herein, we simplified this arterial-to-venous transfer of biomarkers by assuming a homogeneous distribution of the biomarker in the systemic circulation, and that the volume of this idealized vascular compartment was equal to the total volemia. An additional consideration was made for the cerebral circulation, where permeability across the BBB was incorporated as a governing factor to free diffusion of brain-specific biomarkers. This dynamic range theoretically extends from a biomarker diffusivity (cm^2^/s) of zero to a diffusivity that equals the concentration-driven diffusion of a given molecule in bodily fluids. This spectrum of values is biologically unrealistic, but was established for convenience (see also Eq. 3 and paragraphs below). The extent of “opening” for the BBB was based on clinical observations (see Fig. 2b), and the kinetic property of molecule extravasation was based on empirical results (see Fig. 2a) [4, 5, 17, 18]. While there is a large difference between measurements based on contrast-enhancement versus diffusion of a molecule from brain to blood, we suggest that this “Radiologic Index” is currently the best comparative approach to model the behavior of a diffusible marker against clinically acceptable means. Please note that markers’ concentrations in the blood were set to 0 ng/ml at the beginning of the simulation so that a kinetic progression toward steady-state levels could be observed.

**Fig. 1:**
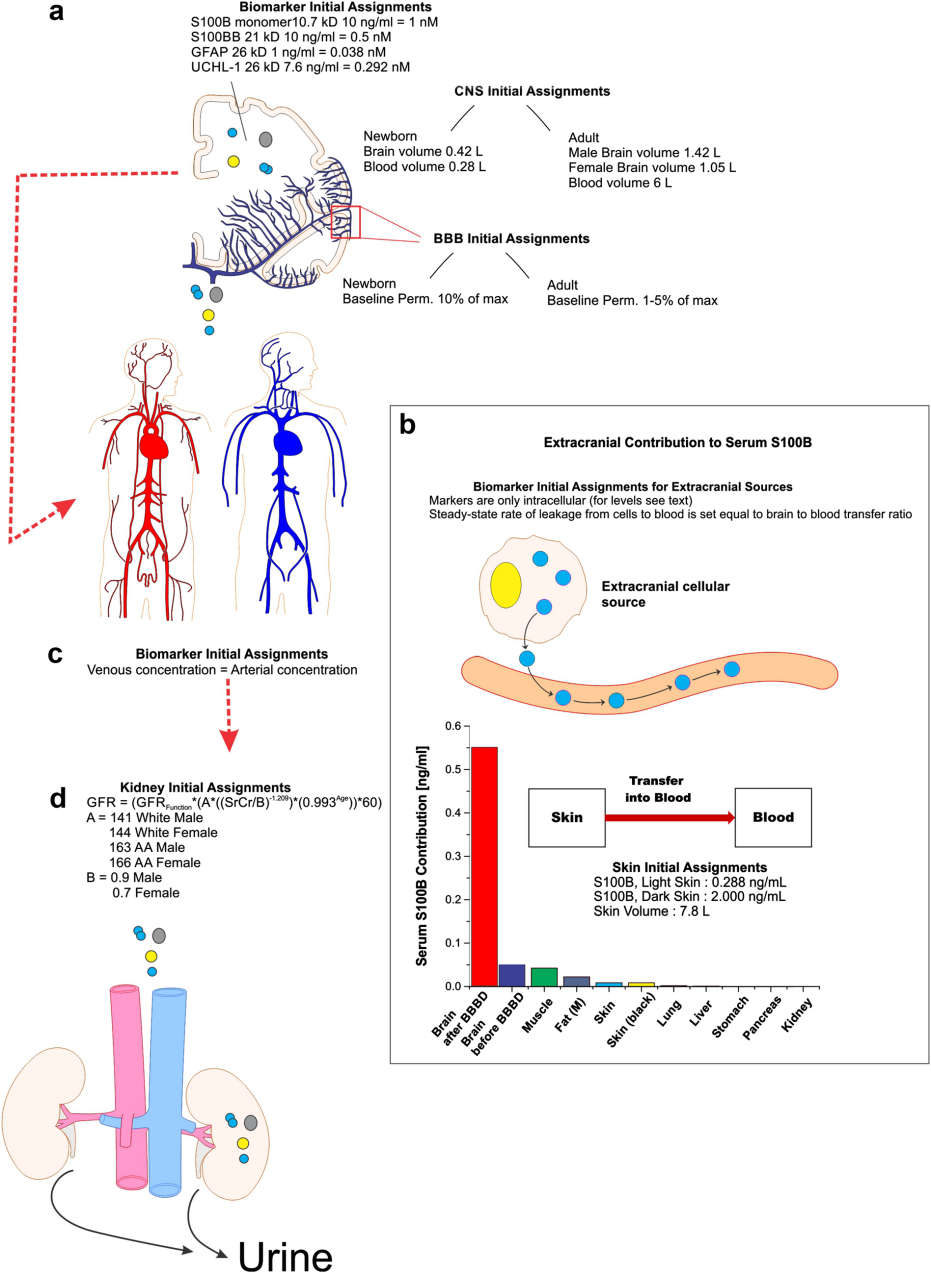
Initial assignments and assumptions for the pharmacokinetic model. The illustrations provide a region-specific grouping of all initial assignments and assumptions considered in our kinetic modeling of biomarker distribution. A detailed graphic and mathematical description of the model is in Additional file 2: Figure S2. Parameters incorporated into the CNS **a** included: (1) molecular weight and concentration of biomarkers; (2) neonatal brain volume and volemia; (3) adult male/female brain volume and volemia; and (4) homeostatic (pre-BBBD) permeability levels across the BBB (see “Methods” section). Extracranial contributions to serum biomarker levels **b** do not significantly differ from a model whose only contribution comes from the brain. Extracranial sources of S100B were quantified using data from [11], and each organ was set to a fixed (1–5%) rate of marker’s transfer to blood. The corresponding* bar plot* shows organ-specific contribution to serum levels. The flowchart in the inset shows a simplified diagram of the skin-to-blood contribution of S100B in the pharmacokinetic model. Arterial and venous blood volumes were combined into a common, systemic blood compartment **c** and an assumption of homogeneity was employed for serum biomarker levels. The blood compartment was provided an initial biomarker concentration of 0 ng/ml. Passage of biomarker mass into the kidneys **d** was dependent on initial assignment of glomerular filtration rate (GFR), as calculated by the Cockroft-Gault formula for both African American (A–A) and Caucasian male and female adults. Neonatal kidney filtration was preset to 47 ml/min/1.73 m^2^ (see Table 1)

Biological markers, as the ones modeled in this manuscript, are present in different CNS compartments. For example, S100B and GFAP are expressed at high levels in astrocytes (but not neurons or other brain cell types) but can also be detected in CSF as well as in interstitial fluid (ISF). Since the kinetics governing intracellular-to-extracellular exchange for these biomarkers is poorly understood, we used clinically available data to assign each biomarker an initial brain concentration (Fig. 1). The initial assignments used reflect what can be measured in extracellular fluid in normal brain. In spite of this simplification, our approach and modeling allow to replicate the common features of many neurological diseases (i.e., gliosis) if the increase in marker’s source concentration can be estimated or measured. Gliosis is a secondary sequela of many acute injuries such as TBI, stroke, etc. During the gliotic process, GFAP and S100B are increased in astrocytes as well as in ISF and CSF.

### Model background

**Fig. 2:**
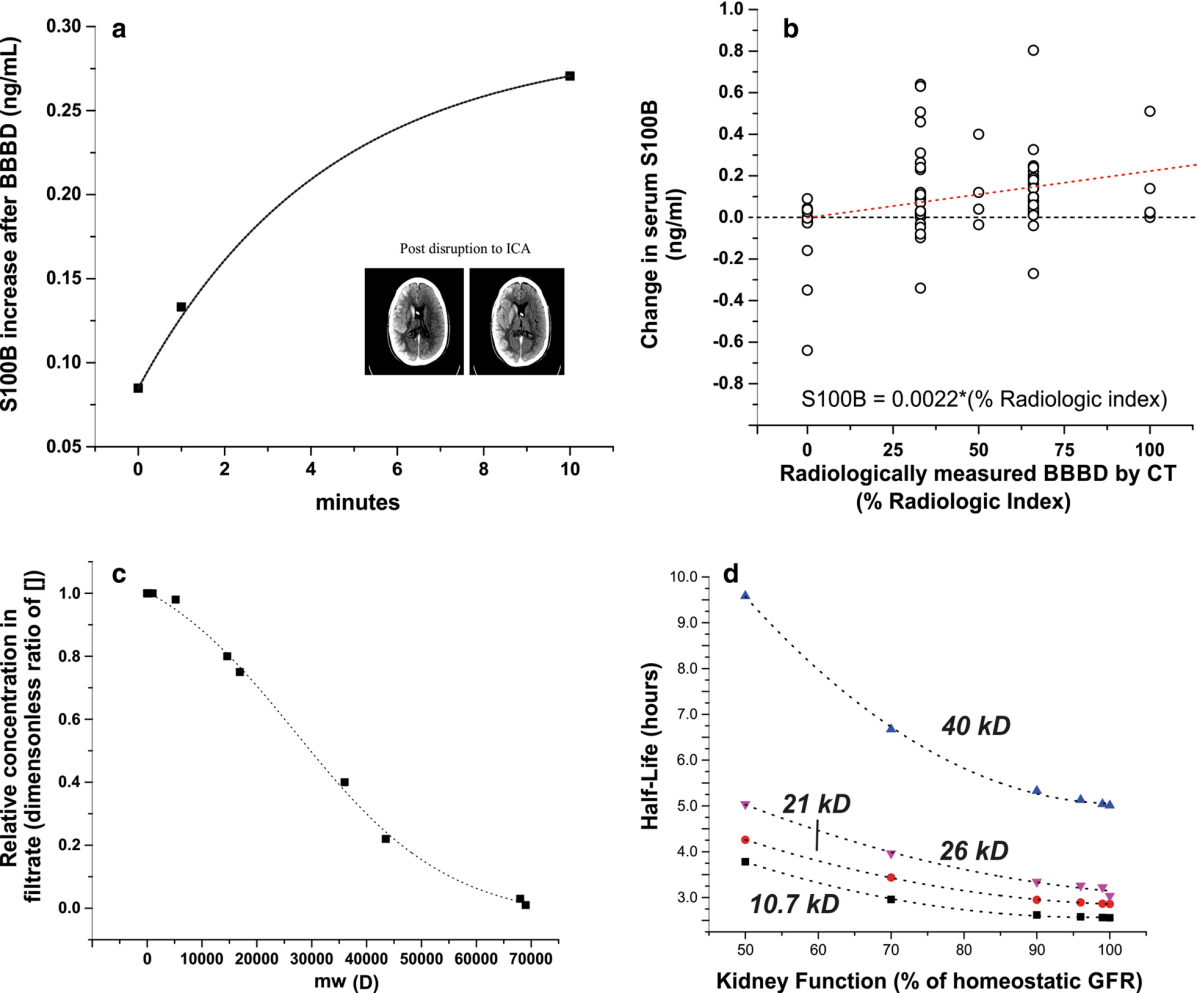
Experimental and theoretical determination of blood–brain barrier characteristics, and quantitative assessment of the effects of biomarker molecular weight on modeling results. The kinetics of BBBD in this model were derived from data from previous studies that involved human patients receiving iatrogenic osmotic opening of the barrier. Time-dependent opening of the BBB was modeled in accordance with **a**, Eq. 2 which shows the time course of serum S100B elevation after intra-arterial infusion with 1.6 M mannitol. The extent at which serum S100B levels were affected by BBBD was modeled in accordance with (**b**, Eq. 3, see *dashed red line*); a radiologic scale of BBB opening shows that 0% BBBD promotes no change in serum S100B, while maximal BBBD causes an increase of ~0.22 ng/ml in serum S100B. Note the* dashed black line* indicating no change in S100B to show that when a BBB disruption >25%, most changes in S100B levels were positive. For details regarding procedures in **a** and **b**, see “Methods” section. The *inset* in **a** shows an example of contrast-enhanced CT imaging used to quantify BBBD. In this case, the hyperosmotic mannitol solution was perfused through the internal carotid artery (ICA). In addition to glomerular filtration rate, a biomarker’s Filtration Coefficient (C_F_) determines the rate at which a marker is cleared through the kidneys (**c**, Eq. 5), with proteins of higher molecular weight having a lower turnover rate from blood into urine. *Figure* **d** demonstrates the dependency of biomarker half-life on molecular weight

We used available data from patients undergoing BBBD by osmotic means [5, 17, 18] to determine the rate of S100B increase in blood. The time-dependent data corresponding to sudden increases in S100B for these patients was fitted to Eq. 3:

3 $$ \left[ {{\text{S1}}00{\text{B}}} \right]_{\text{serum}} = \, 0. 2 9- 0. 20*0. 7 9^{\text{time}} $$

where time is expressed in minutes after the osmotic shock. For details see [5] and Fig. 2a.

Cross-validation of “goodness of BBB opening” measured by peripheral S100B and CT enhancement was performed as described [17, 18]. Maximal osmotic and bi-hemispheric BBBD was set as 100% while no effect of BBBD was computed as 0%. S100B was measured at time of imaging by contrast CT and plotted in Fig. 2b as the difference between post- and-pre-disruption S100B values in serum. In the model, we expressed the time-dependent change in BBB permeability according to Eq. 3 and the subsequent change in blood S100B according to Eq. 4. We assumed in the simulation a steady-state, physiological “leak” of S100B across a healthy BBB as 1–5% of maximal possible hemispheric disruption, as per Eq. 4:

4 $$ \left[ {{\text{S1}}00{\text{B}}} \right]_{\text{serum}} = \, 0.00 2 2*\left[ {\text{Radiologic Index}} \right] $$

The relationship between molecular weight (MW) of a biomarker and its propensity to be filtered by the kidneys, referred to herein as the filtration coefficient (C_F_), was based on Eq. 5:

5 $$ {\text{C}}_{\text{F}} = \, \left( { - 0.0 40 9 4 { } + \, \left( { 1. 1 9 6 1 4} \right)/\left( { 1 { } + { 1}0^{{\left( {\left( { 2 70 9 6- {\text{MW}}} \right) \, * \, - 3. 1 {\text{E}} - 5} \right)}} } \right)} \right) $$

where the value of C_F_ falls between 0 (no filtration) and 1.0 (complete filtration). Empirical data used to create this fitted equation was obtained from [19]. A graphic description of the model is provided in Additional file 1: Figure S1.

### BBB disruption in patients

All patients signed an informed consent according to institutional review protocols of The Cleveland Clinic Foundation and the Declaration of Helsinki. Eight patients with the histologically-proven, non-acquired immunodeficiency syndrome Primary Central Nervous System lymphoma (PCNSL) consented to participate in an institutional, review board-approved protocol for the management of this disease at the Cleveland Clinic Foundation. This protocol involved the concurrent administration of intravenous chemotherapy and a treatment that included BBB disruption [20] followed by the instillation of intra-arterial chemotherapy (IAC). This subset of patients also agreed to additional blood draws for serum S100B sampling. The appropriate inclusion and exclusion of patients on this protocol was documented previously [21]. Specifically, these patients were treated with intra-arterial injection of mannitol causing a temporary disruption of the BBB, followed by a selective, intra-carotid chemotherapeutic injection. The procedure consisted of the following steps: (1) patient is taken to the operating room and general thiopental anesthesia is induced; (2) catheterization of a selected intracranial artery (either an internal carotid or vertebral artery) is performed via a percutaneous, trans-femoral puncture on a given treatment day; (3) mannitol (25%; osmolarity 1372) is administered intra-arterially via the catheter at a predetermined rate of 3–12 cc/s for 30 s; (4) after the BBB is “opened” with mannitol, intra-arterial methotrexate is infused. Immediately following delivery of chemotherapy, non-ionic contrast dye is given intravenously; (5) the patient is transported, still anesthetized, for a CT scan. This step is essential to determine and document the extent of BBB opening since better disruption portends better delivery of chemotherapeutic drugs across the barrier. Methods for grading the degree of BBBD and correlation of these grades with Hounsfield units were previously described [22]; degree of BBBD was graded by visual inspection as nil, fair, good, or excellent; (6) after the CT scan is completed the patient is awakened, extubated and monitored in the hospital overnight. Blood samples were drawn 10 min prior to mannitol injection and 2–5 min after mannitol injection. S100B was measured on all available blood samples by techniques described elsewhere [5, 13]. A total of 102 BBBD procedures in eight patients were studied. The results in Fig. 1 refer to 14 procedures consisting of intra-arterial chemotherapy not preceded by BBBD.

### Serum S100B measurements

Serum samples of S100B were obtained after induction of anesthesia, immediately prior to and immediately after intra-arterial mannitol infusion (Fig. 2a). At each time point, blood samples were collected and immediately centrifuged at 1200×*g* for 10 min, and the supernatant sera were stored at −80 °C. The S100B concentration was measured by the Sangtec 100 ELISA method (Diasorin, Stillwater, MN) using high and low level manufacturer-provided controls to ensure proper assay performance. A total of 267 apparently healthy subjects were prospectively enrolled in compliance with IRB regulations. Serum samples were collected in different seasons (summer and winter), from different regions of the USA (North, Central, and South), and of light and dark skin color. Dark skin color was defined according to FDA guidance (“dark skinned” is defined as Black or African–American, “light skinned” is defined as White, Hispanic, Asian, American Indian, Alaska Native, Native Hawaiian, and other Pacific Islander).

## Results

### Age-related differences in blood biomarkers dynamics

**Fig. 3:**
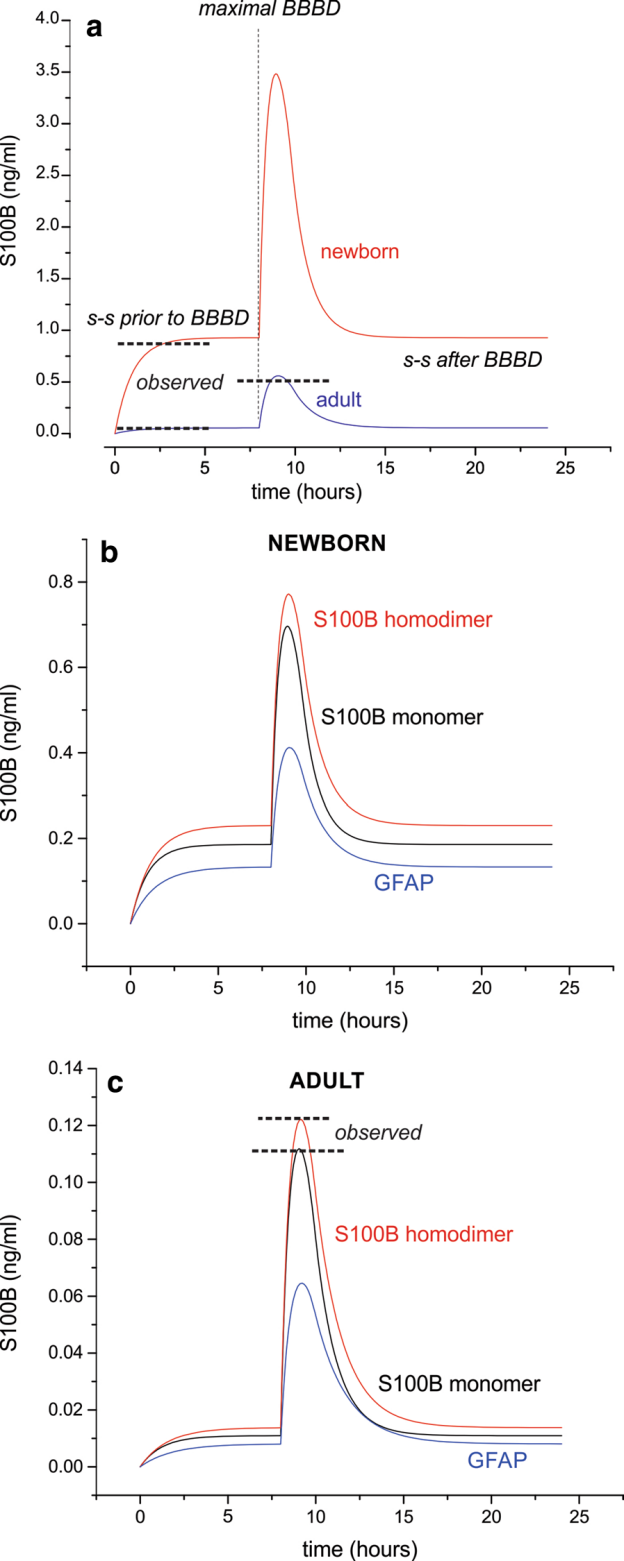
Predicted differences in biomarker kinetics between neonates and adults, based on GFR, body size, and steady-state BBB function. The plot shown in (**a**) demonstrates, for steady-state S100B levels in blood, a ~16-fold increase for newborns compared to adults (0.92 and 0.055 ng/ml, respectively). After maximal BBBD, newborns presented a more dramatic increase in serum S100B concentrations. The *horizontal dashed lines* in (**a**) show a consistency between the observed levels and results from prior literature, for steady-state as well as maximal BBBD in adults [3, 24, 40]. *Figure* **b** and **c** show the behavior for serum levels of the homodimeric form of S100B (21 kD), as well as GFAP (26 kD) and S100B monomer. The concentration profiles in a newborn **b** show a significantly increased steady-state and post-BBBD serum level for all biomarkers, compared to an adult (**c**). The differences among markers within a neonatal or adult population was entirely attributed in our model to GFR values. The *horizontal dashed lines* in **c** again show consistency between model predictions and results from previous studies [3, 24, 40]

Since the model we developed encompasses several features of human physiology that are age-and-biomarker-dependent, we first analyzed the effects of age on serum values for biomarkers of varying molecular weight (MW). To our knowledge, data on UCHL-1 and GFAP levels in healthy newborns are not available, so we instead used S100B values which have been reported to decrease from an average of 0.9 to 0.3 ng/ml in the first postnatal months and further decrease to 0.11 ng/ml in adolescence [23]. For healthy adults, S100B levels in serum are below 0.1–0.12 ng/ml [3, 24, 25]. Of the physiological variables that may contribute to different biomarker concentrations between newborns and adults, we focused on three possible, non-mutually exclusive factors: (1) GFR is significantly lower in the neonatal stage of development, and does not reach fully mature levels until after infancy; (2) body size, and specifically the ratio of brain volume to volemia/body weight, is dramatically increased in babies; and (3) homeostatic BBB function may differ post-gestation compared to adulthood. The results of the modeling, and any discrepancy between experimental data and model results, are shown in Fig. 3a–c. The plot in Fig. 3a shows steady-state and BBBD-triggered changes in serum S100B for a newborn with a brain-to-blood volume ratio of 1.5 (0.42:0.28 l), compared to a ratio of 0.2 (1.4:6.0 l) for adults. This model also incorporated reference values for both neonatal and adult GFR which have been previously reported [12, 26–28]. For details regarding these parameters and other initial assignments, see Fig. 1 and Table 1. The simulation was run as follows: we initially started with a level of 0 ng/ml for serum biomarker and observed an initial progression toward steady-state, which varies based on age-specific variables. After steady-state was established we simulated a maximal BBBD (see *vertical dashed line* in Fig. 3a), which gradually decreased to represent a time-dependent recovery of BBB integrity, and the return of leakage rates to steady-state levels. Serum biomarker levels decreased to steady-state at a rate dependent upon kidney function and therefore the MW of the biomarker. Note that newborn steady-state levels of S100B prior to BBBD were significantly elevated compared to that of a healthy adult. Similarly, the extent of the maximal BBBD-induced serum increase for S100B was exaggerated in the newborn. The *horizontal dashed lines* in Fig. 3a and c emphasize the strong correlation between experimental results and output of the model. Note the excellent agreement between predicted S100B values at pre-BBBD steady-state and the results of the model.

Since one of our goals was to expand this model to include other markers, we added a variable that takes into account protein excretion, at a given GFR, for different MWs. The results are shown in Fig. 3b and c while Eq. 5 shows the modeling relationship used to extrapolate kidney filtration for a marker’s MW. In newborn (Fig. 3b), the steady-state and post-BBBD values for two brain markers with different MWs are shown alongside the kinetic curves of monomeric vs. homodimeric S100B [29]. Note that increased MW resulted in pronounced increases in clearance time, which translated into longer persistence of the signals. Similar results were obtained in adults (Fig. 3c). Please note that, although neonates and adults were modeled using physiological values for body size and kidney function, the initial concentration of brain markers in neonates was set equal to adults. These results emphasize how age-related differences in steady-state and post-BBBD serum levels of each marker may be explained by anatomic (e.g., brain volume) or physiological (e.g., steady-state BBB permeability) variations.

### Gender-related differences in blood biomarker levels

This model predicted minimal physiological changes in serum biomarker levels between an adult male and female. This is consistent with previously reported data showing no gender-specific variations in steady-state levels of S100B [30]. Although the Cockroft–Gault formula for estimating glomerular filtration rate provides a lower rate of elimination for females than males, extent of contribution by the brain is also decreased due to a smaller brain-to-blood volumetric ratio [31]. This deviation from the physiology of the adult male resulted in a slightly varied kinetic curve, due to reduced clearance of biomarkers from female subjects’ serum. The difference predicted by the model is not clinically relevant as gender-driven differences have not been reported.

### Ethnicity-related differences in blood biomarker levels

**Fig. 4:**
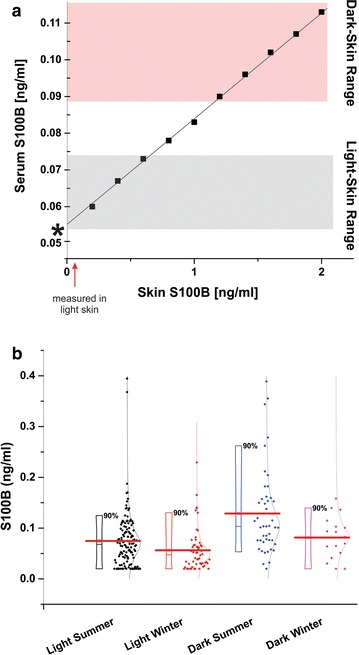
Predicted differences in serum S100B levels as a result of skin pigmentation. **a** When the initial parameters shown in Fig. 2 (*insert*) were used, these parameters predicted a serum S100B level of 0.065 ng/ml for light-skinned subjects, which is comparable to previously recorded findings within this subpopulation (*asterisk* near axis). Note that we used realistic level for skin S100B, which was taken from our previous study and the data in Fig. 2. In order to output accurate serum S100B levels for dark-skinned subjects, the model required that we increase skin concentration of S100B to above 2.0 ng/ml, which resulted in a serum concentration of 0.115 ng/ml. This implies that any change in a subject’s skin pigmentation (e.g., tanning) will increase levels of S100B. This was experimentally confirmed in **b** showing the results of a comparative analysis on the effects of exposure to sun. Note the significant increase in S100B after sun exposure regardless of whether dark skinned (Latinos, African–American subjects) or light skinned individuals were studied

Recent literature has demonstrated a clinically relevant difference in serum S100B levels based on race and regional/seasonal variance, where individuals of a darker complexion have been reported to have higher steady-state S100B levels than those of lighter complexion (i.e., Caucasians during summer compared to winter in the Northern hemisphere [32], or individuals of African–American (A–A) compared to Caucasian descent [30, 33]). It was initially believed that ethnicity is the main driving force for elevated S100B in African–American subjects [30, 33]. If this were the case, based on available GFR data [12], our model would predict a *lower* biomarker level in this population due to increased clearance. Since this is obviously not the true reason for the observed elevations in steady-state levels, we added a skin compartment to the model to predict the following: (1) the contribution of dermal tissue to S100B levels for a given biomarker present in dermal tissue, at steady-state tissue-to-blood transfer rates (2% of maximal), and (2) sensitivity of this contribution value to changes in dermal biomarker tissue concentrations (Fig. 4). We also measured S100B in serum of 267 apparently healthy subjects in different seasons (summer and winter), regions of the USA (North, Central, and South), and in light or dark skinned individuals as described in “Methods” section. This was done to test the hypothesis that varied levels of sun exposure are sufficient to account for the differences originally attributed to ethnic factors. The initial assignment of skin S100B concentration in light-skinned subjects was derived from a previous study of organ-specific S100B levels, which indicated that brain tissue has a 34.7:1 concentration ratio with skin. This initial value was accompanied by a set secretion rate equaling 2% of free diffusion for a small molecule, a rate corresponding to that of the BBB under steady-state conditions.

An obvious limitation of this approach is that one needs to input an initial concentration for dermal S100B or any other organ contributing to serum levels. We therefore measured levels of S100B by ELISA in freshly resected surgical samples from normal access tissue (Fig. 1) and these values were added to an appropriate volume of skin [14]. Only adult males were considered for this portion of the simulation. The results confirmed our hypothesis: when using the measured values of skin [S100B] and the appropriate volumetric ratios, the model accurately predicted increases in serum S100B based on sun exposure or skin pigmentation differences due to race. Note that sun exposure resulted in different levels of S100B even within a light (or dark) skinned population. Unlike in the modeling results presented in Fig. 3, changes in BBBD-induced S100B were minimally effected (*not shown*). This is to be expected, given that BBBD only effects cerebral vasculature permeability.

## Discussion

The main outcome of this study was the implementation of a MatLab-based pharmacokinetic model that allows to study or interpret the fate and excretion, levels and half-life of markers derived from the CNS but sampled in the blood compartment. A corollary set of hypotheses, which were largely confirmed by cross-validation of the model with existing data, implicated the variation of markers’ levels due to: (1) physiological parameters (e.g., GFR); (2) somatic properties (volumetric size of different organs during development); and (3) environmental factors such as sun exposure.

### Strengths of the model

One of the key strengths of this model, and the results presented herein, is the extent to which these results can be validated by empirical data. These data were primarily obtained from our own work but we also used findings by others in the public domain. In addition, we used a realistic model of the human body, based on the success of PBPK analysis of drug AMDE [14]. In these models, and in the variation adopted by us, the body is represented as a network of intercommunicating compartments; each organ has an adjustable volume to accommodate anatomical variations, and the organs are interconnected by a realistic vascular tree with arteries and veins. However, the capillary compartment is not included.

The main strength and uniqueness of this approach resides in the clinical data we used to model permeability of the blood-brain barrier. Our results are based on uncommon inter-arterial procedures used to treat brain neoplasms. For details and rationale of this procedure, see [20]. Pertinent to this effort is the fact that “opening” of the BBB was clinically measured at time of blood testing by contrast-enhanced CT scans. Figure 2b shows the quantitative relationship between radiological measurements of BBBD and associated changes in blood S100B. Please note that because of the clinical nature of this trial and the large number of subjects enrolled, the data are not as clear-cut as one desires. Human studies were still utilized over available data from animal studies, however, due to increased translatability and clinical relevance.

Another significant feature of our modeling effort is the presence of excretive systems. This may come as a surprise given that the main focus of our research is in neurosciences. However, the modeling results demonstrate that one of the chief regulators of markers’ presence in blood is the level of GFR. We were able to show that kidney function (both physiologic and pathologic; Fig. 2d) also affects markers’ half-life in a size-dependent manner. In other words, with physiologic kidney function, half-life was linearly related to markers’ molecular size. However, when approaching kidney failure, the effect was overwhelmingly shifted toward markers with higher (over 40 kD) molecular weight. This is important because markers of brain and BBB damage can be very small (S100B, 10 kD), of intermediate size (tau, 46 kD), or large (autoreactive IgG, 140 kD). We underscore that without adjusting for molecular weight and kidney function, one may misinterpret the true clinical meaning of a given marker. For example, if one wishes to determine the delayed sequelae of a given event (e.g., stroke, TBI) it is best to use a marker with a longer half-life (higher molecular weight).

An additional aspect that we wish to discuss is the use of accepted values for the markers’ initial levels in the brain (Fig. 1a). We also modeled the relative changes in brain-to-blood volume due to changes in age and gender, as well as extracranial biomarker sources. In the case of S100B, it is widely reported that skin and fat contain substantial levels of S100B [10, 34]. In our model we used measured values for fat and skin S100B content (Fig. 1b). By doing so, we were able to show that skin levels directly affect steady-state serum S100B levels, and what is more important, they also reproduce changes in basal S100B levels due to ethnicity, exposure to sun and skin complexion. As in the other modeling efforts, we used real data to confirm or disprove the output of the model (Fig. 4). Fat tissue, when measured in a broad range of BMI, has been reported not to influence blood S100B [11]. This may be surprising since the measured levels of S100B in skin were in fact lower than levels in fat. This discrepancy can be explained by two mechanisms, namely the high cellular turnover and death rate of dermal cells [9] and the poor vascularization of adipose tissue compared to dermal tissue [35].

In every modeling effort, the source of modeling inputs is essential. Despite our efforts to use meaningful input values, some aspects of this approach require further studies to improve output accuracy. For example, MRI is the recognized quantitative tool to measure BBBD and yet we used CT. This was due to the fact that, at the time of our experiments, not only was intraoperative MRI not available, the velocity of acquisition in CT scanning made their use more amenable for fast-paced, intra-arterial procedures. Furthermore, the length of time required for MRI signal acquisition was inconsistent with the time resolution required by the model (minutes, see Fig. 2a).

Another limitation of this approach is the fact that the transfer of intracellular markers to the extracellular space is not fully understood, and certainly not known for the biomarkers discussed herein. We used as a surrogate for the movements of S100B across the plasma membrane data from melanoma cell lines expressing high levels of S100B (see Additional file 1: Figure S1 and Reference [9]). However, since none of the markers studied or modeled appear to have endocrine or exocrine functions, we believe it is safe to assume that their rate of intracellular-to-extracellular transfer is low in healthy tissue. By the same token, it is reasonable to predict that physical trauma will mobilize the marker from soft tissues such as skin and fat, and that under condition of traumatic events, the contribution of extracranial sources may well be different than at steady-state. In addition, while every effort was made to use available knowledge on brain and body development and aging, we lacked quantitative values for the brain concentration of various biomarkers in the newborn population.

## Conclusions

In conclusion, we developed a multi-compartment, pharmacokinetic model that integrates the biophysical properties of a given brain molecule and predicts its time-dependent concentration in blood, for populations of varying physical and anatomical characteristics.

## Additional files

**Additional file 1: Figure S1.** Graphic depiction of the model use in the simulations described herein.

**Additional file 2: Figure S2.** Mathematical modeling of the kinetic properties of biomarker release from astrocytes. The data were modeled using the data in [9]. The underlying assumptions made in this Figure and in Reference [9]: S100B release is shown in the case of cellular damage (symbolized by the “hole” in the plasma membrane. Leakage of S100B or GFAP by other means and across an intact cellular membrane has not been described but cannot be ruled out. Levels of S100B release are expressed as fM per cell/h, which can be used for future modeling efforts. Whether the same applies to GFAP, another astrocytic protein, is unknown.

## Abbreviations

CNS: central nervous system
MHI: mild head injury
TBI: traumatic brain injury
BBB: blood–brain barrier
GFAP: brain-derived glial fibrillary acidic protein
UCHL-1: Ubiquitin C-Terminal Hydrolase L1
AMDE: absorption, distribution, metabolism, and excretion
