# Supplementary figures and images for: Improving the clinical management of traumatic brain injury through the pharmacokinetic modeling of peripheral blood biomarkers
Source: Fluids Barriers CNS. 2016 Nov 30;13:21. doi: 10.1186/s12987-016-0045-y (PMC5402680; doi:10.1186/s12987-016-0045-y)

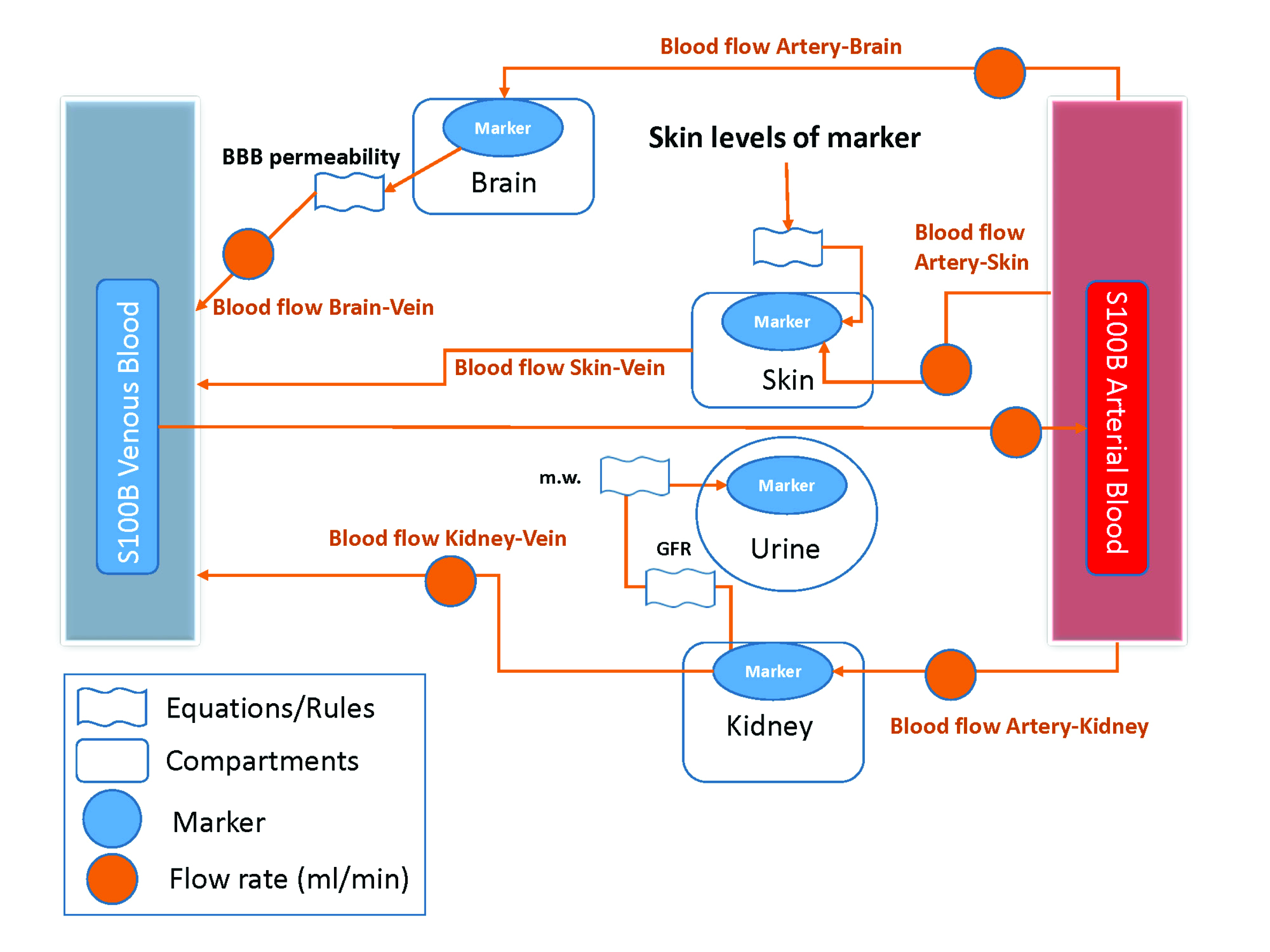

Supplement: Supplementary file 1 — Additional file 1: Figure S1. Graphic depiction of the model use in the simulations described herein. [file 12987_2016_45_MOESM1_ESM.jpg]

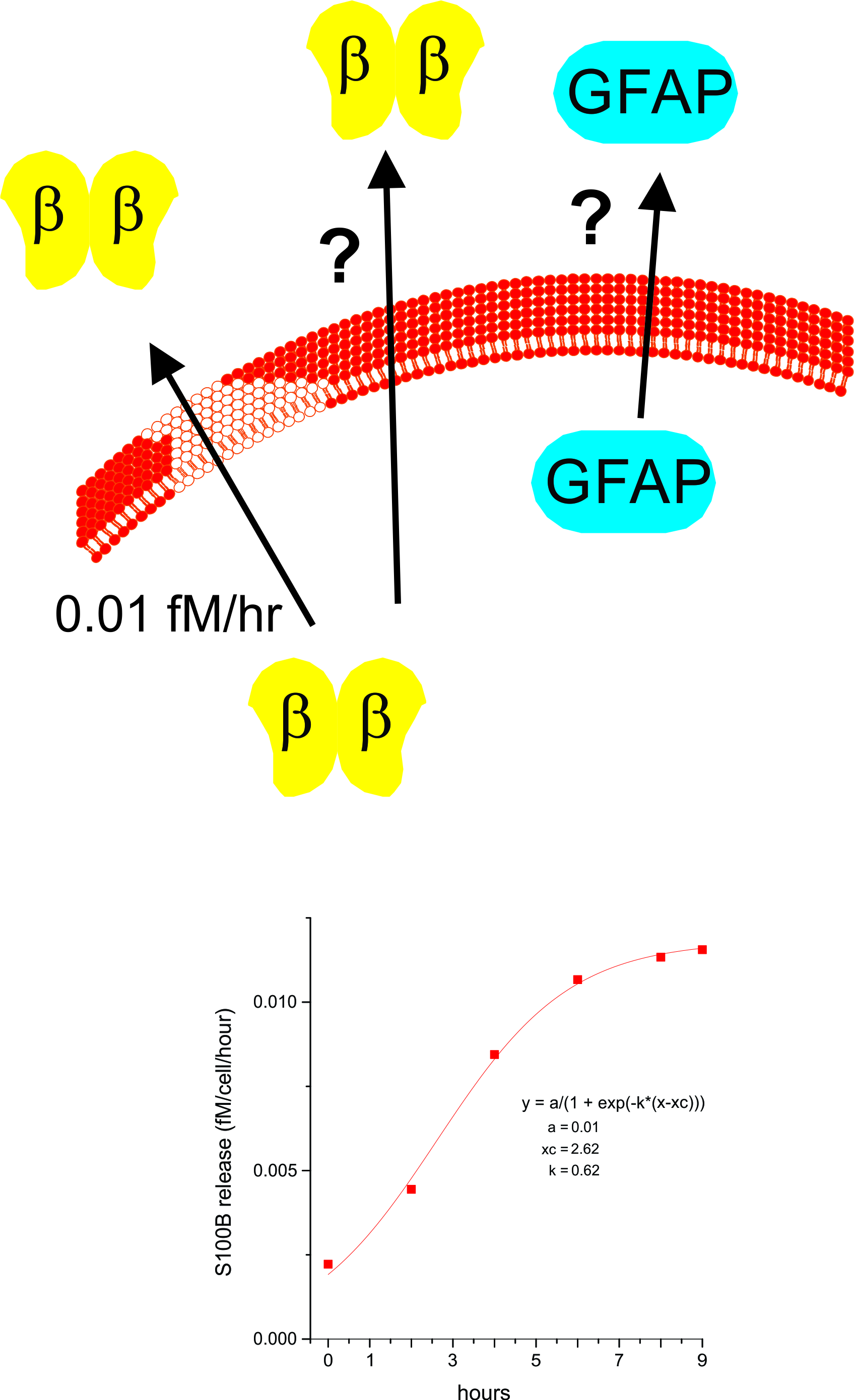

Supplement: Supplementary file 2 — Additional file 2: Figure S2. Mathematical modeling of the kinetic properties of biomarker release from astrocytes. The data were modeled using the data in [9]. The underlying assumptions made in this Figure and in Reference [9]: S100B release is shown in the case of cellular damage (symbolized by the “hole” in the plasma membrane. Leakage of S100B or GFAP by other means and across an intact cellular membrane has not been described but cannot be ruled out. Levels of S100B release are expressed as fM per cell/h, which can be used for future modeling efforts. Whether the same applies to GFAP, another astrocytic protein, is unknown. [file 12987_2016_45_MOESM2_ESM.jpg]
